# ASSOCIATION BETWEEN NEIGHBORHOOD ENVIRONMENT AND FEAR OF FALLING AND FALLS AMONG COMMUNITY-DWELLING OLDER ADULTS

**DOI:** 10.1093/geroni/igad104.0017

**Published:** 2023-12-21

**Authors:** Pamela Dunlap, Xiaonan Zhu, Jennifer Brach, Kyle Moored, Alyson Harding, Andrea Rosso

**Affiliations:** University of Pittsburgh, Pittsburgh, Pennsylvania, United States; University of Pittsburgh, Pittsburgh, Pennsylvania, United States; University of Pittsburgh, Pittsburgh, Pennsylvania, United States; Johns Hopkins Bloomberg School of Public Health, Baltimore, Maryland, United States; University of Minnesota School of Public Health, Minneapolis, Minnesota, United States; University of Pittsburgh, Pittsburgh, Pennsylvania, United States

## Abstract

Neighborhood built environment may influence both objective and perceived risk of falling. We examined the association between neighborhood built environmental characteristics and falls, fear of falling (FOF), and gait efficacy among older adults. We used baseline data from a randomized trial that measured falls over the past 12 months, FOF, and Modified Gait Efficacy Scale (mGES). Walkability audits were conducted for participant home addresses using Google StreetView and summarized by two factors: urbanicity and neighborhood quality. Logistic and linear regression were adjusted for age, gender, race, comorbidity index, and body mass index. Analyses were also stratified by past falls and gait speed (< or ≥1.0m/s). We included 249 participants (mean age: 77.4 years; 65% female) with 29.7% reporting a fall over the past year and 40.6% reporting FOF. Mean mGES (0-100) was 85.2±13.7 and 32% participants had gait speed <1.0m/s. In unstratified analysis, greater urbanicity was associated with lower gait efficacy (β=-1.88, p=.039). Among those who had not fallen, greater urbanicity was associated with FOF (β=0.47, p=.012) and greater urbanicity and higher neighborhood quality were associated with lower gait efficacy (β=-2.8, p=.006; β=-2.1, p=.047). Among those with better mobility, urbanicity was associated with lower gait efficacy and FOF (β=-2.4, p=.009; β=0.5, p=.01). There were no significant associations among those who had fallen or with poorer mobility. Neighborhood environmental characteristics may play a larger role in FOF among those who have better mobility and have not yet fallen, as they may have more opportunities to interact with varied environmental conditions.

